# A virtual experiment on pedestrian destination choice: the role of schedules, the environment and behavioural categories

**DOI:** 10.1098/rsos.211982

**Published:** 2022-07-27

**Authors:** Christopher King, N. W. F. Bode

**Affiliations:** Department of Engineering Mathematics, University of Bristol, University Walk, Ada Lovelace Building, Bristol BS8 1TW, UK

**Keywords:** virtual experiment, pedestrian dynamics, destination choice, statistical model calibration

## Abstract

Which locations pedestrians decide to visit and in what order drives circulation patterns in pedestrian infrastructure. Destination choice is understood to arise from individuals trading off different factors, such as the proximity and busyness of destinations. Here, a virtual experiment is used to investigate whether this behaviour depends on the layout of buildings, whether planned or imposed destination schedules influence decisions and whether it is possible to distinguish different choice behaviour strategies in pedestrian populations. Findings suggest that virtual experiments can consistently elicit a range of destination choice behaviours indicating the flexibility of this experimental paradigm. The experimental approach facilitates changing the environment layout while controlling for other factors and illustrates this in itself can be important in determining destination choice. Destination schedules are found to be relevant both when imposed or generated by individuals, but adherence to them varies across individuals and depends on prevailing environmental conditions, such as destination busyness. Different destination choice behaviour strategies can be identified, but their properties are sensitive to the detection methods used, and it is suggested such behaviour classification should be informed by specific use-cases. It is suggested that these contributions present useful starting points for future research into pedestrian destination choice.

## 1. Introduction

Pedestrian destination choice describes the behavioural process by which people decide on the locations they would like to walk to and in what order. It has been described as a strategic level process, distinguished from tactical level processes, such as route choice, and operational level processes, such as collision avoidance [1]. Adopting the formalism of transportation, it could be described as the mechanism by which origin–destination matrices are formed and potentially change over time. Thus, pedestrian destination choice is immediately relevant for pedestrian flow in buildings, event sites and cities, making it crucial for understanding pedestrian traffic.

The broad mechanism for pedestrian destination choice is considered to be based on individuals trading off different factors against each other when making decisions (e.g. [1–3]). For example, pedestrians who seek to complete an activity at a destination quickly may want to avoid busy destinations, and, to minimize their effort, they may generally prefer nearby destinations. Research on this topic is therefore concerned with determining which factors are relevant and how important they are in determining the destination choices of individuals. The two predominant types of evidence used are surveys and direct observations. Surveys, in which individuals state their choices retrospectively or state their intentions, have been used as a means to investigate the choice behaviour of pedestrians for the last couple of decades (e.g. [4–8]). The motivation for these studies is primarily economic, investigating contexts such as shopping or tourist movement behaviour. Direct observation relies on recording the movement of many individuals using dedicated active sensing, such as mobile phone applets [9], or passive sensing, such as recording the physical location of portable devices using Wi-Fi signals (e.g. [2,10]). While their usefulness is evident, both data collection approaches have drawbacks. For example, surveys either rely on the memory of individuals or hypothetical situations, and in direct observations, it can be challenging during busy periods, where crowd densities are high, and movement speeds are slower, on average, to determine whether individuals are visiting a destination or are simply passing through it. An alternative and comparatively infrequently used approach to obtain evidence on pedestrian destination choice is controlled virtual experiments where individuals interact with a carefully controlled simulated environment. While the ecological validity of such experiments needs to be examined carefully [11], the fact that the information provided to participants can be controlled precisely makes it possible to directly investigate aspects of pedestrian destination choice that are difficult to manipulate in stated choice surveys or direct observations.

A ubiquitous feature in pedestrian destination choice research is the intrinsic motivation of pedestrians to visit a destination. Itineraries or schedules capture an order of ‘priority’ for activities pedestrians want to complete at different destinations [12], and they thus influence their destination choice behaviour. The desirability of destination has been investigated in different ways, notably in market research [13,14]. Sigmoid functions have been used to express the preferences of individuals for destinations [15], and features of buildings [2,16] or the number of transitions between destinations [17] have been used to estimate such preferences. In this contribution, a virtual experiment is used to explore how schedules influence destination choice and if there is a difference between imposed schedules and ones created by individuals.

There are two broad and related methodological questions that need to be considered in pedestrian destination choice research. The first question is concerned with the extent to which destination choice behaviour can be generalized. For example, if data are recorded in one building, to what extent can the findings be applied to another building. To the best of the authors' knowledge, no work quantitatively investigating the potential differences in choice behaviour has been attempted to date. Related to the first question, the second question is concerned with the extent to which people exhibit different behaviour in surveys or laboratory experiments than in reality (i.e. this evidence lacks ‘ecological validity’). Previous work investigating and quantifying potential differences in participant behaviour due to different data collection methods has been studied in the field of economics [18,19]. Mahmassani & Jou [19] compare commuter travel behaviour from a hypothetical scenario presented in a laboratory with travel diaries collected in a field study. They fit a route choice model to data from each collection method and find that the models contain the same parameters, and each parameter has the same sign; however, the actual estimated values were different. Other studies investigate the effect of the data collection method on participant behaviour, but while they compare the advantages and disadvantages of different data collection methods, no quantitative effects are measured (e.g. [20]). Here, the flexibility virtual experiments offer is used to measure how differences in the environment (building layout) and in the way information is presented change choice behaviour. Therefore, this work takes exploratory steps to address the questions above in the context of pedestrian destination choice behaviour.

It has been suggested that in many contexts, people can be separated into distinct groups according to the behaviours they display, including movement patterns [4,5,21–24], activity patterns [25–27] and mentality [6,7,28]. Clustering procedures either make use of author-defined summary statistics of the data [5,24,26], or use algorithms, such as unsupervised machine learning [22,23,28], spatial density methods [4,21] and sequence alignment [25,27], based on features of the quantities measured. Currently, statistical model calibration is used to fit and explain the data collected using a specified model. To the best of the author's knowledge, no clustering based on model calibration has been attempted. This paper therefore attempts to demonstrate a new application of statistical model calibration through cluster individuals based on the destination choice behaviour.

To summarize, the following questions are answered and formally assessed through hypothesis tests:

1) Do people use a mental schedule of destinations that they plan to visit?
2) Do people make different decisions if they are recommended an order to visit destinations, compared with choosing their own?
3) Does the layout of the environment influence pedestrian destination choice behaviour?

While initial results are provided for the following questions, which are also further discussed in §4:

i) Can a virtual experiment elicit consistent destination choice behaviour regarding how distance and occupancy affect the destination choices made by people?
ii) If schedules are used, how strictly do people follow them?
iii) Does the way in which information is presented affect peoples' choices?
iv) Can people be categorized in terms of their choice behaviour through model calibration? If so, how does it compare with clustering through traditional methods?

The remainder of this manuscript is structured as follows. Section 2 discusses the experimental design (§2.1), the recruitment methods used (§2.2) and subsequent data analysis (§2.3.1), including data clustering methods (§2.3.2). Results are presented in §3, including characteristics of the participant samples (§3.1), model estimation (§3.2) and participant clustering (§3.3). The implications of the results, along with ideas for future work are discussed in §4.

## 2. Methodology

### 2.1. Experimental design

In the virtual experiment, participants were tasked with choosing a sequence of destinations within a fixed time period. Instead of explicitly simulating the movement of individuals, participants were asked to choose destinations consecutively with their positions and remaining time being updated instantaneously between decisions.

**Figure 1.**
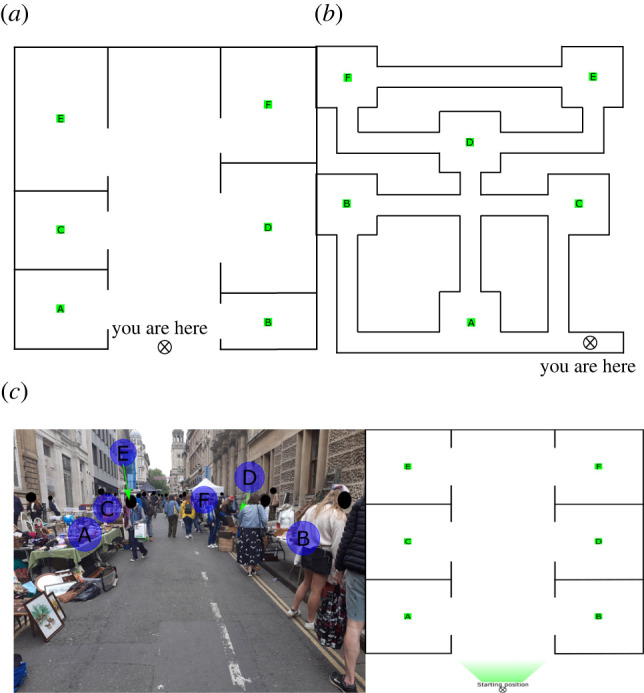
The different virtual environments used: the open environment (*a*), the closed environment (*b*) and the open environment with information about destinations given in the form of first-person photographs (*c*). A map of the environment showing the position and perspective of the photo is also provided. These images are presented to participants at the start of the experiment.

Three different virtual environments and visualizations were employed and these are shown in figure 1. Each environment has six destinations, distinguished by letters A–F. Figure 1*a* shows the 'open environment’, where there is an open space between destinations, making it is easy to move between any two destinations. Figure 1*b* represents the ‘closed environment’, where movement between destinations is restricted by passageways. The final environment in figure 1*c* was visualized differently to the preceding environments. Instead of showing an abstracted top-down view, it shows a real-world representation of an environment similar to the open environment. Here, information on each destination is shown through photographs taken from a first-person perspective. For clarity, a diagram illustrating the points-of-view of the photographs accompanies them. The images displayed during the photo survey questions are shown in appendix D. Sample screenshots of the different stages of each experimental condition are provided in appendix C.

The experiment was conducted online using an online survey platform^1^ which allowed for response-based routing. All experimental conditions shared the same basic structure. First, the purpose of the experiment was explained (see figure 6). Second, participants had to consent to taking part in the research (figure 7). If the participant did not agree to take part, then they were taken to a final page which again gave them the opportunity to take part in case they changed their mind. If the participant agreed, then they were asked for some basic information: their age, their gender/sex and the kind of environment they lived in (for details see §3.1). These questions were optional and were included to ascertain how representative of the general population the sample of participants was. Investigating the possible effects of these demographic measures was not a main interest of this work, and the explanatory power of measures, such as self-reported gender or age, can be compounded by other factors. Next, participants were provided with instructions and information about the task. Each experimental condition was designed to take 3–4 min to complete. The schedule chosen condition type was estimated to take a little longer to complete than the others, due to the additional task of creating a schedule of destinations to visit. Ethics approval for the experiment was obtained from the Faculty Research Ethics Committee in Engineering at the University of Bristol.

In each experimental condition, the participants had to complete the same basic task—conduct a five-destination trip in the displayed environment within a set hypothetical time. The trip consists of up to five choices of destination made by participants using information available in an accompanying image of the environment. The image displayed shows the participant their current location, the locations of other destinations and the occupancy of other destinations. The current location of a participant in the environment is either the initial position if the participant is starting their trip, or the destination which they decided to visit in their previous choice. The environmental layout gives participants a rough idea of the relative distance between destinations. Occupancy of destinations is represented by blue circles, with each circle representing an individual using the destination. The experiment ended either when participants ran out of hypothetical time or when they had selected a sequence of five destinations. In either case, the choices they made were recorded and used in subsequent analyses.

A hypothetical time limit is employed to provide realistic consequences for participants choosing busy and/or further away destinations. For the photo condition, the hypothetical time limit for completing the trip is 15 min, which was chosen as a realistic time to move in the environment and visit five destinations. For the other experimental conditions, the hypothetical time limit was arbitrarily chosen to be 60 min, as the environment images give no sense of spatial scale. When participants made a choice, the hypothetical time limit was reduced by a given amount according to the destination chosen. The hypothetical time elapsed depends on both the distance of the chosen destination from the participant's current position and the occupancy of the destinations using the following relation:

2.1 $$T_{i}(j)=\;w_{d}(j)d_{i,j}+\;w_{o}o_{i,j}+\;\varepsilon ,$$

where *T_i_*(*j*) is the time elapsed from the outcome of the *j*th choice being destination *i* from the set of all other destinations not previously visited. *d_i_*_,*j*_ is the distance between the participant's current position and *i*. This distance is measured in arbitrary units and is arbitrarily scaled such that $d_{i,j}\in Z,d_{i,j}\in [0,10]$. The value of *d_i_*_,*j*_ is chosen based on the environment layout, if two destinations are further apart in the image, then the distance between them is higher. The distances for the photo and open environments are equal, but the distances for the closed environment are different from these, due to the different environment layout. *o_i_*_,*j*_ is the current occupancy of *i*, the total number of people present at destination *i*, at the time of the *j*th choice, such that $o_{i,j}\in Z,o_{i,j}>\;0$. For the open and closed environments, the occupancy of each destination changes each time a choice is made, and these changes in occupancies were set by the experimenters to ensure a representative range of occupancies for each destination (see appendix A for details). However, the virtual environment is not simulated dynamically, meaning that if two participants make the same sequence of decisions, they see exactly the same destination occupancy. These systematic changes in occupancy were used due to limitations in survey implementation. For the photo condition, destination occupancy is determined from the number of people present at each destination and so does not change over each choice. The occupancy of all destinations for each environment is shown in appendix A. *w_d_* and *w_o_* are constants which weight the influence of distance and occupancy on *T_i_*, respectively, $w_{d},w_{o}\in R,w_{d},\;w_{o}>0$. Values of *w_d_* and *w_o_* are shown for each environment in appendix B and are chosen such that the number of possible chosen destination combinations for each experimental condition is implemented on the online survey platform manageably while still being reasonably large. Participants are not aware of equation (2.1), only that choosing destinations that are further away and/or are busier will cause more time to elapse. $\epsilon ∼N(\mu ,\sigma^{2})$, the parameters depend on which environment is used. This adds random noise to the otherwise deterministic relationship to prevent any participants from working out the relationship between *T_i_*, *d_i_* and *o_i_*. For the photo environment, ε ∼ *N*(1.5, 0.75), the other environments use ε ∼ *N*(0, 2). This is because the total time and elapsed times in the photo environment are much smaller than in the other environments and using these distributions prevents *T_i_* < 0. Importantly, as the virtual environments are not simulated dynamically, as mentioned above, the remaining times for each possible participant trajectory are only computed once and then used throughout the experiment and for all participants. The time remaining after making each choice is shown to participants when they make their next choice. If participants run out of hypothetical time before making five choices, then the experiment ends by telling the participants that they have run out of time and have had to cut their trip short.

For all experimental conditions, participants were not allowed to re-visit destinations. The reason for this is that in most real situations pedestrians will rarely visit the same destination twice in one trip. They may pass through the destination in order to visit other destinations, but this does not count as a choice to visit the transient destination (e.g. in the closed environment).

In the schedule chosen condition, participants were asked to plan a sequence of destinations to visit based on the environmental layout and starting position alone. Participants were shown the open environment without other people present and asked to choose which destination they planned to visit first, then the second and so on, until a schedule of five unique destinations was chosen. They were then presented with the populated environment and asked to choose their destinations in the same way as the other experimental conditions.

In the schedule given condition, a suggested sequence of destinations to visit is given in the initial information before participants make any choices. The suggested schedule was chosen to be ‘DEBAC’, which did not minimize or maximize the total distance travelled or the occupancy of chosen destinations. However, if participants chose to follow this sequence, then they would run out of hypothetical time.

The photo experimental condition uses photographs of an outdoor flea market in Bristol, UK, which took place on Saturday 19 June 2021. The photos were taken by the authors between 14.30 and 15.30. This environment was chosen as it closely resembled the open environment (figure 1*a*) in terms of layout and destination positions. Destinations were chosen to be stalls positioned on each side of the street that were clearly visible from the initial position. It also allowed occupancies of each destination to be seen from every other destination, allowing participants to use occupancy information in their decisions. First, a photo was taken from an initial position starting at one end of the street, corresponding to the initial position in the open environment condition. Then, photos were taken from the location of each destination, giving views of each other destination. This allowed participants to choose their next destination from their current destination using information from all other destinations. To help participants understand from what perspectives each photo was taken, the photos were numbered, and a diagram is shown alongside the photos, with numbered arrows pointing along the rough line-of-sight of each photo taken. The diagram also shows the participant's current location in the environment.

In order to address our research questions, five different experimental conditions were considered:

1) Base case—this acts as the reference case for comparison. It uses the open environment shown in figure 1*a* and does not involve schedules.
2) Schedule chosen—before choosing which destinations to visit, participants are required to create a schedule of the destinations they would like to visit and in what order.
3) Schedule given—participants are provided with a suggested order of destinations to visit in the environment.
4) Closed environment—the environment used is that shown in figure 1*b*.
5) Photo—the information on each destination in the environment is provided by photos taken from a human observer's perspective (figure 1*c*).

### 2.2. Participant recruitment

To recruit participants, the authors posted messages on various social media, such as LinkedIn and ResearchGate^2^ advertising the experiment, along with messages within their own personal and professional networks. Additionally, participants were recruited from within the University of Bristol by use of internal mailing lists. No remuneration was provided to participants, as each version of the survey took only a couple of minutes to complete. The participant recruitment was designed to reach a large audience that may be sympathetic to contribution to research without remuneration.

The recruitment messages contained a link to a landing page. When opened, this page contained a link that randomly selected one of the five experimental conditions when clicked. Participants then worked through the experiment task, either successfully or unsuccessfully completing it within the set time. If a participant was unsuccessful, then they had the option to re-take the experiment by clicking a link. This option was added based on feedback from the experiment prototypes. The link took them to a copy of their original experimental condition, where data was recorded and stored separately to distinguish it from their first attempt. Regardless of whether a participant was successful in completing the task, an invitation to take part in another experimental condition was provided at the end. This invitation contained a link to a second landing page, identical to the one linked in the original recruitment message. As for the first landing page, participants were randomly allocated one of the five experimental conditions. However, this landing page linked to copies of the five experimental conditions, where data was recorded separately from the first attempts. Each respondent was assigned only one experimental condition for each attempt. This separation procedure was implemented to help account for the learning effect from participants taking part more than once, seeing that for privacy the identity of participants was not recorded.

Before data collection commenced, criteria for terminating data collection were set. It was decided that the minimum number of first attempts needed would be 10 for each experimental condition (50 people in total) and the maximum number would be around 150 per experimental condition (750 total). Data collection was scheduled to take place over eight weeks, or until the maximum number of participants was reached, whichever came first. The experiment was released on 9 July 2021 and was closed on 20 July due to a high response rate.

### 2.3. Choice model

In order to quantify the choices made by participants, a discrete choice modelling framework is used. Discrete choice models are used to estimate the probability of an entity making a choice from a set of distinct, mutually exclusive alternatives [29]. The standard multi-nomial logit model is used, as it is capable of describing a sufficiently broad range of behaviours,

2.2 $$P_{i}=\;\frac{e^{U_{i}}}{\sum_{j\;\in C}e^{U_{j}}},$$

where *P_i_* is the probability of choosing alternative ***i*** from the set of all possible alternatives, *C*, with the denominator acting as a normalization constant. *U_i_* is the utility of alternative *i*. This quantifies the amount gained by the participant by choosing destination *i* and is a function of information available to participants in our virtual experiment.

The utility for the base case, closed environment and photo experimental conditions is a linear function as follows:

2.3 $$U_{i}=\;\beta_{occ}\hat{n_{i}}+\beta_{dist}\;\hat{d_{i}},$$

where $\hat{n_{i}}$ is the normalized occupancy of destination ***i*** at the time the decision is made and $\hat{d_{i}}$ is the normalized distance to destination *i* from the participant's current position. $\beta_{occ}$ and $\beta_{dist}$ are the parameters corresponding to the two predictors. The two predictors are normalized for each decision to between zero and one by dividing each value by the maximum observed value of that predictor at that decision time. This sets a limit for the maximal contribution of each predictor and thus ensures that parameters capture the relative strength of the effects predictors have.

The schedule chosen and schedule given conditions required an additional term to *U_i_*, $\beta_{des}\hat{q_{i}}$, where $\hat{q_{i}}$ is a normalized measure of desire to visit a destination *i* by the participant (defined in more detail below). This is weighted by parameter, $\beta_{des}$.

Both $\beta_{occ}$ and $\beta_{dist}$ can take positive and negative values, representing the potential for occupancy and distance to have an attractive or repulsive effect, respectively. The parameter $\beta_{des}$ can only be positive, because it represents the effect of a participant's chosen or given destination schedule. Negative values would mean that a participant does the opposite to their schedule.

Based on previous work [30], desirability is calculated from a schedule of destinations using the following relation:

2.4 $$q_{k,l}(S_{l})\;=\;e^{-p_{k}},$$

where *q_k_*_,*l*_ is the desirability of destination *k* for participant *l*, *S_l_* is the schedule of destinations for participant *l*. *p_k_* denotes the position of *k* in the schedule of *l*. Destinations further along the participant's schedule are of lower priority than those near the start. If a destination is not in a participant's schedule, then it is given a desirability of zero. When a participant chooses a destination, it is removed from the schedule for all future choices. For illustration suppose, a participant has destination schedule *S* = (A, B, C, D, E) and can choose between the six destinations. So $q=(q_{A},\;q_{B},\;q_{C},\;q_{D},\;q_{E},\;q_{F})=(e^{-1},\;e^{-2},\;e^{-3},\;e^{-4},\;e^{-5},\;e^{-6})$ are the desirabilities for each destination. If the participant in the example visits destination A, their schedule after the visit is now *S* = (B, C, D, E) and $q=(0,\;e^{-1},\;e^{-2},\;e^{-3},\;e^{-4},\;e^{-5})$. Due to the requirement that participants cannot choose to visit a destination that they have previously visited, in practice destination A and therefore *q_A_* would not be considered in future choices.

#### 2.3.1. Data analysis

As mentioned above, data from any additional attempts of the surveys were not analysed as these versions were only included to give participants a chance to retake the experiment without consequence on the original data collected. Investigating learning effects directly was not considered, as there is no way of linking participants between their first or subsequent attempts nor is there a way of determining which experimental condition they did first.

All data analysis was conducted in the R programming environment [31]. First, the sequence of destinations chosen by each participant was extracted, along with demographic information. Frequency distributions of demographic data are reported to assess how representative of the general population the participant sample was. Once the destination sequences were obtained, the distances from the participant's current position and the occupancy of each chosen destination were extracted, given the environment and the choice number using the tables in appendix A. These provide the value for $\hat{d_{i}}$ and ${\hat{n}}_{i}$ in equation (2.3). Additionally, for the schedule chosen and schedule given experimental conditions, the desirability of each destination at each decision is calculated using the schedule chosen by or given to participants, respectively, as described in §2.3.

Next, calibration of the choice model given by equation (2.2) on all responses for each experimental condition is conducted using maximum-likelihood estimation, implemented using the optim function in the R programming environment [31]. To estimate the variance in parameter estimates obtained in this way, 95% confidence intervals are calculated via a bootstrap procedure on the recorded data using 10,000 bootstrap samples.

The parameter estimates are investigated using three hypothesis tests with the following null hypotheses. These hypotheses address the three main research questions in the introduction:

1) The presence of schedules has no significant effect on the choice behaviour of participants.
2) There is no significant difference in choice behaviour between participants of the schedule chosen and schedule given conditions.
3) There is no significant difference in choice behaviour between participants of the base case and closed environment.

To assess hypotheses one and three, permutation tests are performed using the sum-of-square difference of parameter estimates for the two experimental conditions being compared as the test statistic. Permutations construct artificial datasets by randomly re-allocating participants between the two experimental conditions, ensuring that the overall amount of data for each experimental condition remains the same. The proportion of permutations that produce values of the test statistic larger than the one observed in the original data for the two experimental conditions is the *p*-value for the test. The number of permutations for each test is 10,000. To assess hypothesis two, a likelihood-ratio test is performed to assess if including the desirability parameter significantly improves model fit.

#### 2.3.2. Participant clustering

To investigate if different behavioural categories are present in the destination choices of participants in our experiments, clustering methods to separate individuals into groups with similar behaviours are implemented. Numerous methods of data clustering exist, each with their own advantages and disadvantages. To account for this and to demonstrate the importance of methodological choices, two clustering methods are implemented.

The first clustering method is motivated by the possibility that participants may consider one factor, such as distance or occupancy, to be more important. To see whether this is the case, the normalized cumulative distances and occupancies associated with destination choices of each participant for each experimental condition are calculated. The values of these quantities necessarily increase as the chosen destination sequence length increases, so for each participant the cumulative quantities are normalized by dividing by the number of decisions that participant made. The subsequent distributions are examined for signs of multi-modality. If any distributions show two or more modes, then it could indicate the presence of distinct strategies that attempt to minimize/maximize the quantity of interest. A simple way of assigning participants to different clusters is by using threshold values that are the midpoints between two adjacent modes. The destination choice model described in equation (2.3) is then calibrated on each cluster separately and the goodness-of-fit of this clustered model is compared with the model estimated on all participants (aggregate) data using the Akaike information criterion (AIC) [32]. The AIC for a clustered model is the sum of the AICs of the models for each cluster.

The second clustering method is hierarchical clustering [33], as implemented using the default settings of the ‘hclust’ function in R [31]. For the purpose of clustering, the destination choice model is calibrated separately for each individual participant. The estimated parameters are assumed to reflect the destination choice behaviour of participants. The Euclidean distance between the parameter estimates is used for clustering and dendrograms are used to visualize the clustering and to decide on clusters of participants to be examined further by fitting the destination choice model to data from all participants included in the cluster. Model calibration on each cluster is performed and compared with the aggregate model as described above.

#### 2.3.3. Chosen schedule versus chosen sequence comparison

Any differences seen between the schedule of destinations chosen by participants and the actual chosen destination sequences are also investigated. This will show how closely people follow their schedule when they carry out their trip and thus gives an indication of the impact of prevailing conditions, such as destination occupancies, on participant choices.

Quantitative comparison of schedules and chosen sequences is performed using sequence alignment [34]. The length of the chosen sequence can be shorter than the schedule if the participant runs out of (hypothetical) time, so a distance metric that takes deletions and/or additions into account is needed. The simplest distance metric that satisfies these conditions is the Levenshtein distance, which counts the number of operations (substitutions, additions and deletions) needed to convert one string into another.

## 3. Results

### 3.1. Sample statistics

A total of 813 people participated across experimental conditions. Thus, the average number of participants that attempted one experimental condition is 162.6, with the minimum number of participants being 144 for the photo and maximum being 174 for schedule chosen. The average number of participants that attempted one or more experimental conditions two or more times is 53, with the minimum of 49 and the maximum of 57. Of the respondents that chose to repeat their initially assigned experimental condition, the average is 9.4, with the minimum being 6 and the maximum being 16. As stated above, all results presented in this paper are based on first attempts only.

An implementation issue for the base case and closed environment meant that demographic information for participants was not collected, so only demographic information on participants from the other three experimental conditions is reported here.

The majority of participants were between the ages of 18 and 30 (approx. 62%), followed by between 30 and 50 (approx. 26%) and then between 50 and 70 (approx. 11%), with the lowest being people greater than 70 (approx. 1%). No one aged 18 and under took part in the experiment. Only one participant did not answer this question. The estimated average age is 31.9 (3 significant figures (s.f.)) with an estimated standard deviation of 13.7 (3 s.f.).

The majority of participants stated that they lived in a city (approx. 63%), with the next highest number living in a town (approx. 22%), followed by those living in villages (approx. 11%), with those living in hamlets or the countryside giving the lowest proportion (approx. 2%). Only two participants did not answer this question.

There were more female participants than male participants (approx. 55% female, approx. 43% male). Around 1% identified as non-binary and only two people did not answer this question at all. No one answered ‘Other’ for any attempt of any experimental condition.

### 3.2. Model parameter estimation

**Figure 2.**
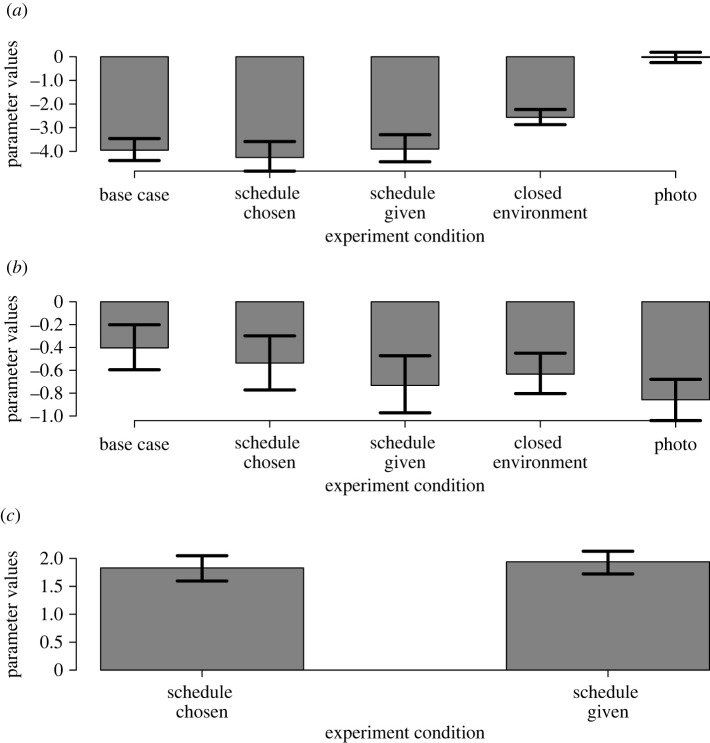
Estimates of destination choice model parameters, *β_occ_* (busyness) (*a*), *β_dist_* (distance) (*b*) and *β_des_* (desire) (*c*) for all experimental conditions. Error bars show 95% bootstrap confidence intervals. These results are also presented in tables 1–5 along with final AIC values for the fitted and random models.

The destination choice model described in equations (2.2) and (2.3) was calibrated on the chosen destination sequences of all participants of each experimental condition separately. That being said, not all participants could be used for the schedule chosen condition, which used only the first 112 out of 174 responses, as issues with data recording meant later responses (after 11.18 on 19 July 2021) had to be discarded. The estimates for model parameters for each experimental condition are shown in figure 2.

**Table 1.** Model-fitting information for the base case experimental condition. Confidence intervals are calculated from 10 000 bootstrap replicates. Point estimates and confidence intervals are given to two decimal places. AIC values are calculated from 794 data points and given to four significant figures.

	point estimate	95% confidence interval
*β_occ_*	−3.95	[−4.39, −3.46]
*β_dist_*	−0.40	[−0.60, −0.20]
fitted AIC	2402	
random AIC	2848

**Table 2.** Model-fitting information for the schedule chosen experimental condition. Confidence intervals are calculated from 10 000 bootstrap replicates. Point estimates and confidence intervals are given to two decimal places. AIC values are calculated from 534 data points and given to four significant figures.

	point estimate	95% confidence interval
*β_occ_*	−4.26	[−4.84, −3.59]
*β_dist_*	−0.54	[−0.77, −0.30]
*β_des_*	1.83	[1.60, 2.05]
fitted AIC	1396	
random AIC	1917

**Table 3.** Model-fitting information for the schedule given experimental condition. Confidence intervals are calculated from 10 000 bootstrap replicates. Point estimates and confidence intervals are given to two decimal places. AIC values are calculated from 774 data points and given to four significant figures.

	point estimate	95% confidence interval
*β_occ_*	−3.90	[−4.44, −3.30]
*β_dist_*	−0.73	[−0.97, −0.47]
*β_des_*	1.94	[1.72, 2.13]
fitted AIC	2035	
random AIC	2777

**Table 4.** Model-fitting information for the closed environment experimental condition. Confidence intervals are calculated from 10 000 bootstrap replicates. Point estimates and confidence intervals are given to two decimal places. AIC values are calculated from 782 data points and given to four significant figures.

	point estimate	95% confidence interval
*β_occ_*	−2.56	[−2.21, −2.79]
*β_dist_*	−0.63	[−0.78, −0.41]
fitted AIC	2568	
random AIC	2805

**Table 5.** Model-fitting information for the photo experimental condition. Confidence intervals are calculated from 10 000 bootstrap replicates. Point estimates and confidence intervals are given to two decimal places. AIC values are calculated from 657 data points and given to four significant figures.

	point estimate	95% confidence interval
*β_occ_*	−0.03	[−0.25, −0.19]
*β_dist_*	−0.86	[−1.04, −0.68]
fitted AIC	2298	
random AIC	2357	

Figure 2*a* shows that the busyness parameter estimates are large and negative for all experimental conditions, except photo. This indicates that, on average, participants were less likely to visit a busier destination than a quieter one. The busyness estimate for the closed environment is lower in absolute value than for the open environment experimental conditions, indicating that occupancy is less important when there are restrictions in travelling between destinations.

Tables 1–5 present the results of fitting the model described in equations (2.2) and (2.3). As the model is fitted to each experimental condition separately, there are five separate tables presented here. The parameter estimates and confidence intervals are also shown in figure 2. To provide a reference AIC value to assess model fit, the AIC of the random model (where all parameters are fixed at zero) is also shown. These results show that the destination choice model used provides a better fit to the data than a random model.

The distance parameter estimates (figure 2*b*) show that destinations further away from the participant's current position are less likely to be chosen, on average. However, the magnitudes of these estimates are significantly less than those of the occupancy parameter, suggesting that distance is less important in the average participant's decision-making process. Again, caution must be taken when interpreting these results, especially when comparing schedule chosen and schedule given with the other experimental conditions, as their destination choice model has three parameters rather than two, and it is the relative contributions of these parameters that matter. Also, the distances in the closed environment are different from those of the open and photo environments, so this must be considered when making comparisons. The qualitative observation that participants on average preferred nearby destinations holds regardless of these considerations.

Figure 2*a,b* helps answer question (i) in §1, suggesting that virtual experiments such as these can produce consistent results as to the relationships between destination choice and potential influencing factors, such as distance and occupancy, at least when these factors are sufficiently controlled. Fortunately, this is easy to do in virtual experiments.

The photo experimental condition gives a busyness parameter estimate close to zero, indicating that occupancy plays no significant role in people's choice of destination when they are presented information in this way. Comparing the distance parameter values between the base case and photo environments show that when destination information is presented as photographs, distance becomes more important. These results together suggest that changing the way in which information about destinations is presented to participants can affect their decisions (question (iii) in §1). However, caution must be taken when making this comparison, as the occupancies for the photo experimental condition are different from those used in the other experimental conditions.

The models including destination preferences according to a schedule have an additional model parameter, desirability. Figure 2*c* shows that, on average, participants are more likely to visit destinations that were present in their schedule. Comparing the magnitudes of the estimates also suggest that the effect of schedules is not affected substantially by whether a schedule is suggested to or chosen by a participant.

The results shown in figure 2 are also used to assess the three hypothesis tests stated in §2.3.1:

1) The presence of schedules has no significant effect on the choice behaviour of participants—rejected at 95% significance ($\chi^{2}(1)\;=\;408$ (3 s.f.), *p* = 1.47 × 10^−59^, likelihood-ratio test).
2) There is no significant difference in choice behaviour between participants of the schedule chosen and schedule given conditions—accepted at 95% significance (*p* = 0.44; permutation tests, *n* = 10 000 permutations).
3) There is no significant difference in choice behaviour between participants of the base case and closed environment—rejected at 95% significance (*p* = 0.0001; permutation tests, *n* = 10 000 permutations).

The results of hypothesis one answer the first main question in §1, showing that people often create a mental schedule of the destinations that they visit and that this can be a significant influence on their destination decisions. Hypothesis two addresses the second main question in §1, indicating that there is no significant effect on the destination choices people make when being able to choose their own schedule versus being provided one to follow. Finally, hypothesis three, which answers the final main question in §1, provides strong evidence that the layout of the environment can have a profound impact on how people decide which destination to visit next.

### 3.3. Participant clustering

**Figure 3.**
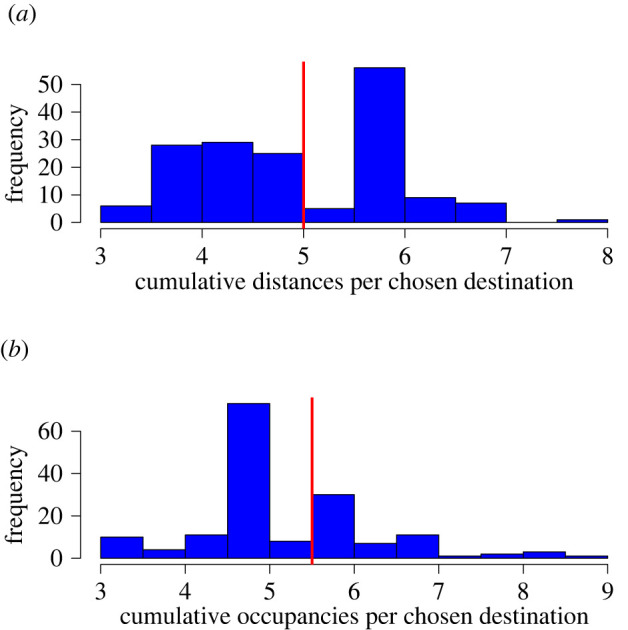
Histograms of normalized cumulative quantities over participants were used to cluster participants of the base case (*a*) and closed environment (*b*) experimental conditions. These quantities are normalized by dividing by the number of destinations chosen by each participant. The red lines show the cluster threshold value for each quantity.

Examining the distributions of normalized cumulative distances and occupancies of participant's chosen destination sequences show that only the cumulative distance in the base case and cumulative occupancy of the closed environment show signs of potential bi-modality (figure 3). This indicates two possible groups of people; those that try to minimize this quantity, and those who do not. The distributions for all other experimental conditions show no multi-modality and are not shown here.

As stated in §2.3.2, the midpoint between the two modes is estimated (shown as a red line in figure 3) and used as the threshold value for assigning participants into clusters. The destination choice model is then calibrated on the clustered data for these two experimental conditions. The results of which are summarized in tables 6 and 7.

**Table 6.** Results from clustering base case participants by normalized cumulative occupancy. Parameter estimates are given to two decimal places.

data	size	*β_occ_*	*β_dist_*
all	166	−3.95	−0.40
considered	88	−2.35	−0.82
travellers	78	−7.09	0.19

**Table 7.** Results from clustering closed environment participants by normalized cumulative distance. Parameter estimates are given to two decimal places.

data	size	*β_occ_*	*β_dist_*
all	161	−2.56	−0.63
extremal	106	−4.76	−1.14
balanced	55	−0.28	−0.30

Both these tables show that the estimates using all participants lie between the corresponding estimates for the two clusters, as expected. Table 6 reveals that the two clusters for the base case are of similar size, with one cluster considering occupancy as detrimental but distance as attractive to destination choice. The positive distance parameter indicates that these participants would rather visit a faraway destination if that destination is quiet, therefore, this group is known as ‘travellers'. The other cluster shows a different strategy, where distance is also detrimental when choosing a destination. This group provides a more balanced attitude towards these factors, so these individuals are part of the ‘considered’ cluster.

Table 7 shows that for the closed environment there are significantly more participants that try to avoid busy destinations more than faraway destinations (‘extremal’). The ‘balanced’ group shows participants who consider occupancy and distance equally when choosing their next destination. The differences in the two tables support the idea that people adopt different choice strategies depending on the layout of the environment.

The AIC for the clustered data is compared with the model calibrated on the aggregated data. For the base case, for the clustered data AIC = 2271 (4 s.f.), and for the aggregated data AIC = 2403 (4 s.f.). For the closed environment, for the clustered data AIC = 2447 (4 s.f.), and for the aggregated data AIC = 2569 (4 s.f.).

**Figure 4.**
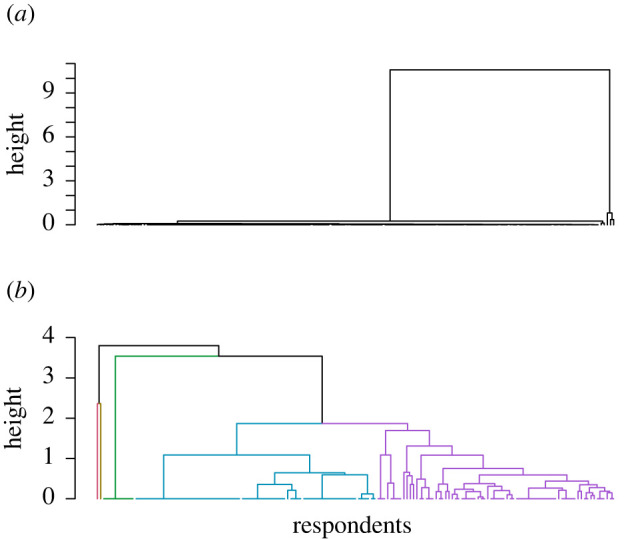
Dendrograms of hierarchical clustering of the base case (*a*) and closed environment (*b*) experimental condition participants using hierarchical clustering of parameter estimates for individual participants. Green = ‘averse’, blue = ‘avoidant’, purple = ‘relative ambivalence’.

Based on hierarchical clustering, only the closed environment (figure 4*b*) shows clear clusters of participants. The remaining experimental conditions give rise to dendrograms similar to that of the base case (figure 4*a*), where the majority of participants are placed into relatively similar clusters, with a few outlying participants.

For the closed environment, some participants are also distinct from the majority of others (shown in brown in figure 4*b*), but there are also three sizable and distinct clusters when the dendrogram is truncated at a height of 1.75 (the green, blue and purple branches of figure 4*b*, representing the ‘averse’, ‘avoidant’ and ‘relative ambivalence’ clusters, respectively). As for the other clustering method, the destination choice model is calibrated on each cluster separately. The results are summarized in table 8. The parameter estimates suggest that participants in each identified cluster prioritize visiting less busy destinations, but to differing extents. These results are quite different from those produced by qualitative clustering in table 7, illustrating the difficulty of finding clear groups of participants and the issues with drawing conclusions based on these groups.

**Table 8.** Results from clustering closed environment participants by hierarchical clustering (figure 4*b*). Parameter estimates are given to two decimal places.

data	size	*β_occ_*	*β_dist_*
all	161	−2.56	−0.63
averse	10	−4.91	−1.66
avoidant	75	−16.81	−2.54
relative ambivalence	74	−0.99	−0.18

The AIC of this clustered model is also compared with that of the model estimated on all data, in this case, for the model fitted to clusters, AIC = 2387 (4 s.f.), and for the model fitted to all data, AIC = 2569 (4 s.f.). By comparing these values with those obtained from the closed environment clusters obtained from the alternative clustering method (see above), it is apparent that clustering through model calibration is a viable way of categorizing different behaviour patterns. Despite producing different clusters, the similarity between the AIC values suggests that the two clustering methods described here produce results of similar quality. The fact that both clustered AICs are smaller than the aggregate AIC indicates that clustering should be attempted in order to obtain a better model fit, with the exact method being at the analyst's discretion and likely to depend on context and requirements for model fitting. This provides an initial answer to question (iv) in §1.

### 3.4. Planned versus chosen destination sequences

**Figure 5.**
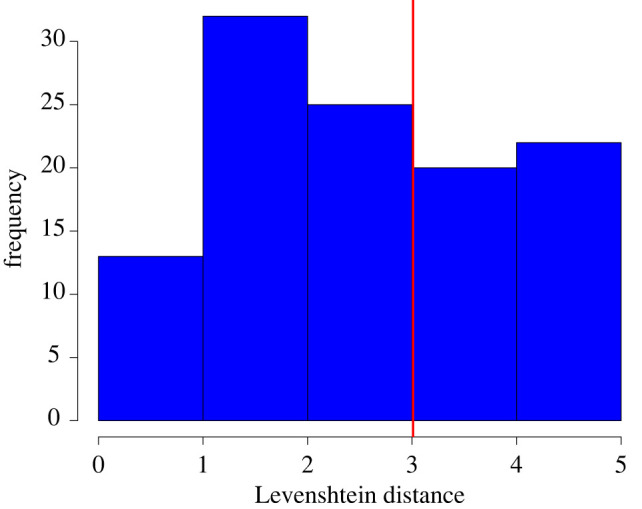
Histogram of Levenshtein distances between schedules chosen by participants and their actual chosen destination sequences. The red line indicates the mean.

**Figure 6.**
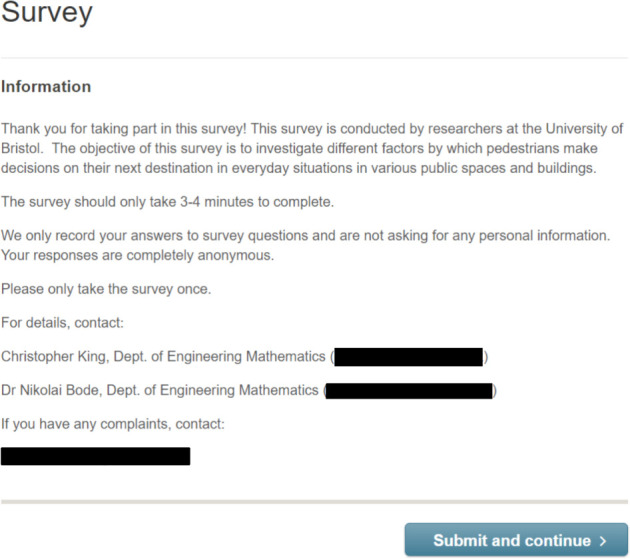
Screenshot of the initial introduction used for the surveys. Participants would then be taken to the informed consent screen shown in figure 7. Actual email addresses have been obfuscated to preserve privacy.

**Figure 7.**
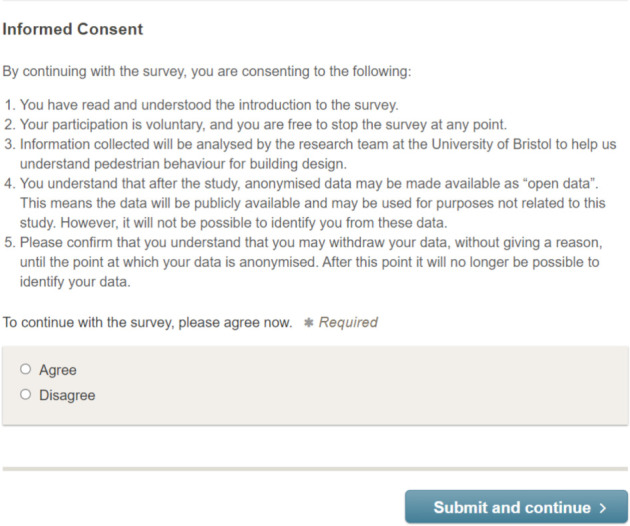
Screenshot of the informed consent used for the surveys. If a participant agreed, then they would continue with the rest of the survey, going to either the instructions in the form of figure 8. If consent is not given, the survey would end.

Figure 5 shows the results from comparing the schedule of destinations chosen by participants before beginning the main experimental task and the actual sequence of chosen destinations as described in §2.3.3. It shows that most people tend to roughly follow their schedule, with only a few changes made. However, there is considerable variation in how much people follow their schedules across participants. Only a few people follow their schedules exactly, indicating that most participants who took part were willing to adapt their planned destination sequence according to prevailing environmental conditions, such as destination occupancies. Therefore, in answer to question (ii) of §1, how strictly a person follows their schedule is dependent on the individual themselves. There is no tendency, at least in the participants studied, for people to follow or disobey their schedule.

## 4. Discussion and future work

This work collects and publishes data on pedestrian destination choice behaviour using a virtual experiment. A simple destination choice model is calibrated on this data, revealing that people tend to avoid busier and further away destinations, with busyness being the more influential factor. These results are consistent across experimental conditions where information is presented identically, showing that virtual experiments can elicit consistent behaviour when occupancy and distance are considered (question (i) in §1). It is also found that the relative importance of these factors can change significantly with environmental layout and how information is presented to participants (questions (3) and (iii) in §1), respectively. Evidence that participants in our experiment can be clustered into different categories according to their behaviour based on model calibration is found and this produces results of similar quality to traditional methods (question (iv) in §1). Finally, results indicate that the majority of participants studied were willing to deviate from their chosen schedule given prevailing environmental conditions (question (ii) in §1).

The first contribution of this work is a demonstration that even a highly abstracted virtual experiment can elicit consistent destination choice behaviour in human participants. As for all such research, the extent to which the behaviours observed also occur in the real world is not clear [11]. Therefore, the specifics of the destination choice behaviour found should not be over-interpreted. In addition, it is possible that the restrictions to social contacts related to the COVID-19 pandemic influenced the tendency of participants to avoid busy destinations in addition to their desire for completing their task within the set time limit. Nevertheless, the findings from this experiment present useful data for comparison with future studies.

The second contribution is that using the high degree of control afforded by virtual experiments, it is possible to compare destination choice behaviour across environmental configurations keeping all other aspects of the experiment and therefore information available to participants the same. While behaviour does not differ qualitatively in that participants avoid busy and faraway destinations in both the open and closed environment, there are quantitative differences in parameter estimates, similar to the results of [19]. This suggests that in applications where the magnitude of destination choice model parameters is important, model calibration may need to be performed separately for different environments. While it must be noted that the distances between destinations in the closed environment are different from those used in the open and photo environments, this was accounted for as much as possible by normalizing distances.

The difference in occupancies between the photo environment and the open environment makes a direct comparison of parameter estimates difficult across these experimental conditions. It should be noted though that the difference in parameter estimates in the photo environment compared with the other experimental conditions indicates that different destination choice behaviours can be elicited depending on what information is presented to participants and how it is presented. To explore the question of the ecological validity of different ways of presenting information more comprehensively would require a comparison of virtual experiments to real-world behaviour or at least experiments conducted in real physical environments (see e.g. [35]). This is left to future work and the data collected here could be used as a starting point for designing such studies.

The third contribution of this work is a demonstration in principle that model calibration can be used to explore data, in this case by identifying different destination choice behaviour strategies can be present in pedestrian populations. This would be expected from previous work suggesting that people can be distinguished into different activity pattern categories, for example [25–27]. However, the comparison of different clustering methods in this work also highlights that the exact nature and composition of any observed behavioural clusters depend on the clustering method used and so caution must be taken when making any conclusions based on these. Nevertheless, this work has demonstrated a new method of clustering individual data, representing another alternative clustering method which could be useful in specific contexts. The most appropriate clustering approach is likely to depend on the specific context and purpose of the clustering. For example, is the intention to improve prediction accuracy of models, or to identify groups of pedestrians with specific predefined behaviours related to how they use a facility, such as a shopping mall or a public transport hub?

Finally, this work shows that a schedule of destinations is likely to influence destination choice, regardless of whether it is imposed or chosen by participants. There is large variation in how different a person's schedule is from their actual chosen sequence of destinations. This suggests that most people are willing to adapt their schedule based on prevailing environmental conditions, such as destination occupancy, and as a consequence that inferring underlying schedules from observed destination sequences would be difficult. Hidden Markov models that have previously been used to infer behavioural states may provide a methodological starting point to infer schedules (see e.g. [36]). In how far knowledge of schedules is important for understanding the dynamics of pedestrian facilities will depend on the context and this presents an interesting topic for future research.

The experimental design could be improved in several ways. Although destination occupancies varied between successive choices, they did not vary over participants, as could be expected in real situations. The implementation also limited the size of the environments and the number of choices made by each participant. These factors could negatively influence the reliability of model calibration, as the parameter space may not be fully explored. The hypothetical time limit for the task can be expected to encourage participants to choose destinations closer and/or less busy to save time, which is likely to restrict the kind of choice behaviour observed. Moreover, the experiment was designed not to depict a specific context, such as suggesting the task was part of a shopping trip or similar, in order to avoid any context-specific behaviours. However, informal feedback from some participants indicated that some people imposed their own context from the environment, which could have influenced the results in unknown ways. Future work could eliminate this potential source of variation by specifying a context in the participant briefing.

## 5. Conclusion

In summary, this contribution demonstrates how highly controllable virtual experiments can be used to contribute to research into pedestrian destination choice. While care should be taken over extrapolating the specific behaviours observed here to pedestrian behaviour in real settings, the principle of some findings is directly relevant beyond the specific experimental setting. First, virtual experiments can be used to elicit different destination choice behaviours suggesting they can be a useful experimental paradigm. Second, the layout of environments itself can be an important factor determining destination choice, even if other aspects are controlled for. Third, destination schedules are relevant when imposed or generated by individuals, but adherence to them varies across individuals and depending on prevailing environmental conditions, such as how busy destinations are. Fourth, different destination choice behaviour strategies can be present in pedestrian populations but methods for detecting them are ideally informed by specific use-cases. These contributions are hoped to present useful starting points for future research into pedestrian destination choice.

## Figures and Tables

**Figure 8.**
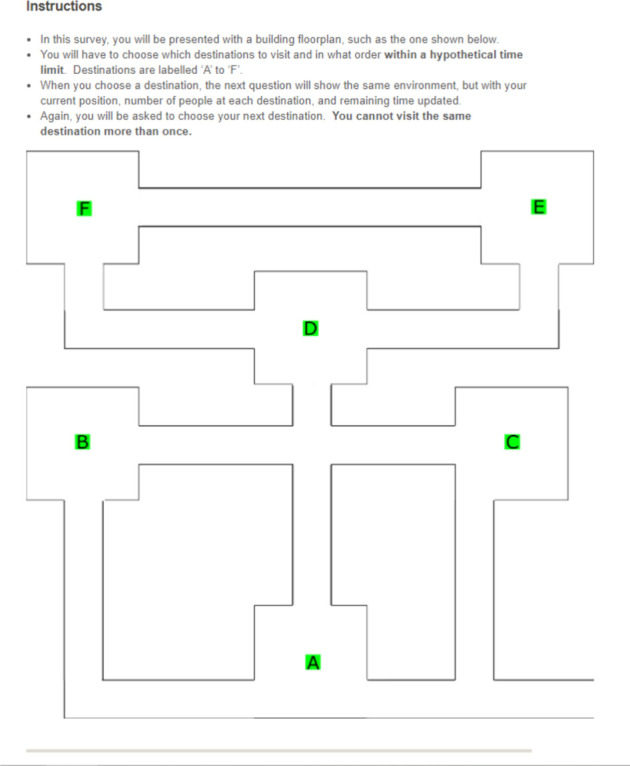
Screenshot of the generic instructions used for all experimental conditions except the photo condition. Continuing would take the participants to figure 9. The instructions for the photo survey differ slightly, describing how information about the environment is presented, see below. Also note that the environment displayed here is different from the environment the participant would be presented later in the survey, i.e. participants of the open environments would be shown the closed environment here, and vice versa for those under the closed environment condition.

**Figure 9.**
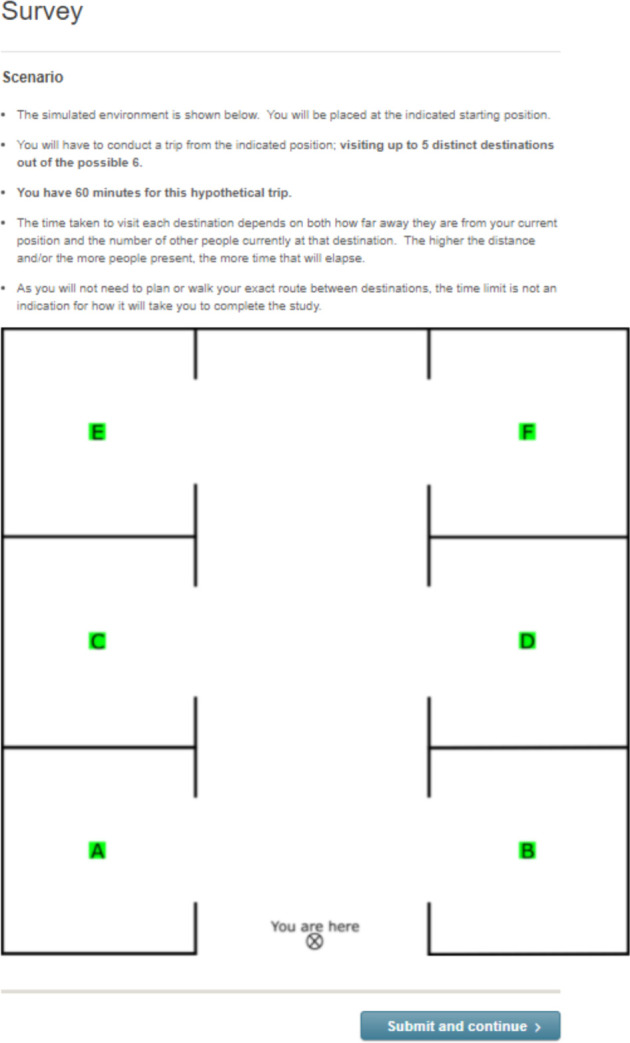
Screenshot of the specific instructions used for all experimental conditions that were not photo or schedule given. The schedule given condition also states the schedule of destinations to visit here. The photo survey shows a photograph instead of the diagram of the environment and the hypothetical time to complete tasks is different, as described in §2.1. Continuing would take the participants to figure 10 for the schedule chosen condition.

**Figure 10.**
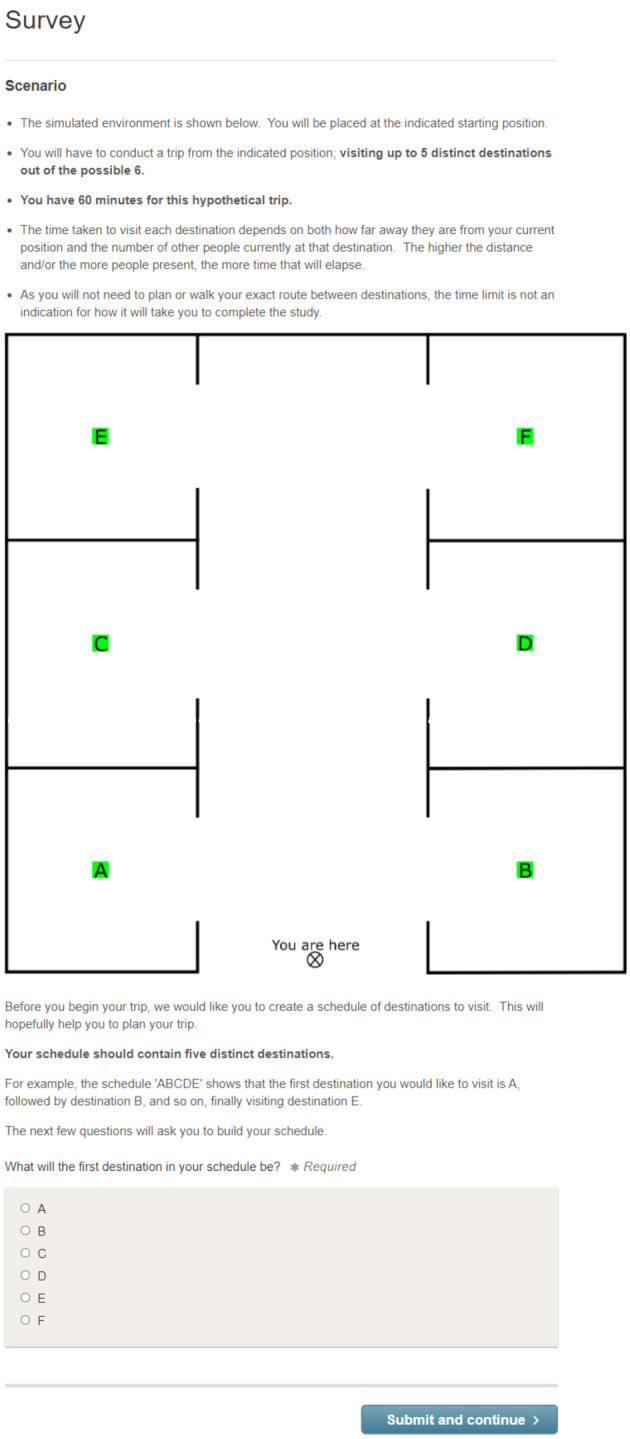
Screenshot of the first question for the schedule chosen condition. Answering the question would take participants to the next question, which would be in the form shown in figure 11. In these questions, the environment pictures are not updated after each submitted answer.

**Figure 11.**
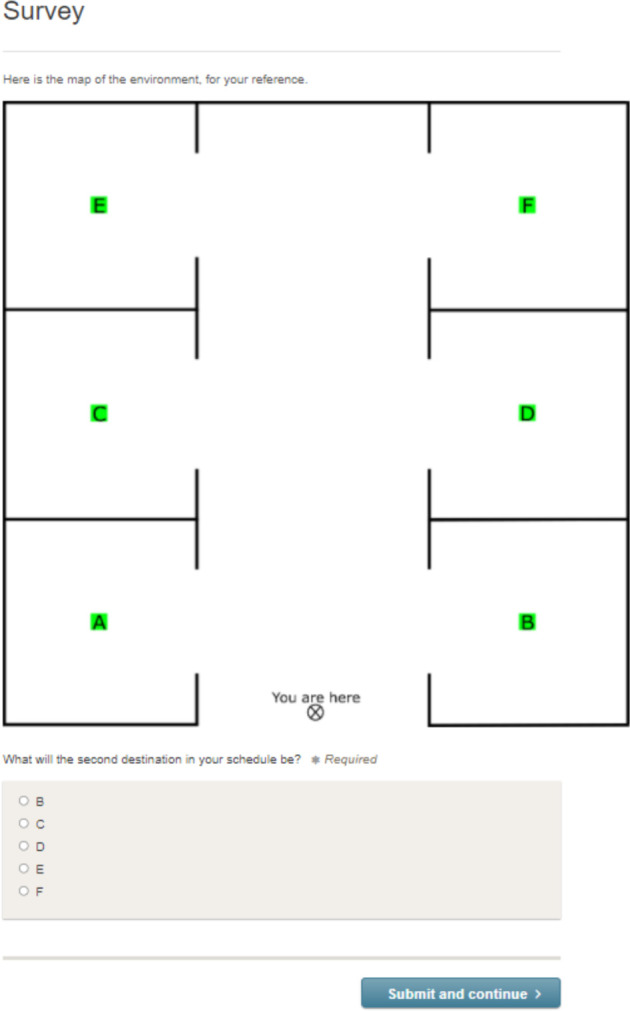
Screenshot of the general form of subsequent questions when choosing a schedule. Participants would be taken to the next question of the same form, but with the responses updated. These questions repeat until the full schedule has been chosen. In these questions, the environment pictures are not updated after each submitted answer. Once the participant has chosen their schedule, they move on to figure 12.

**Figure 12.**
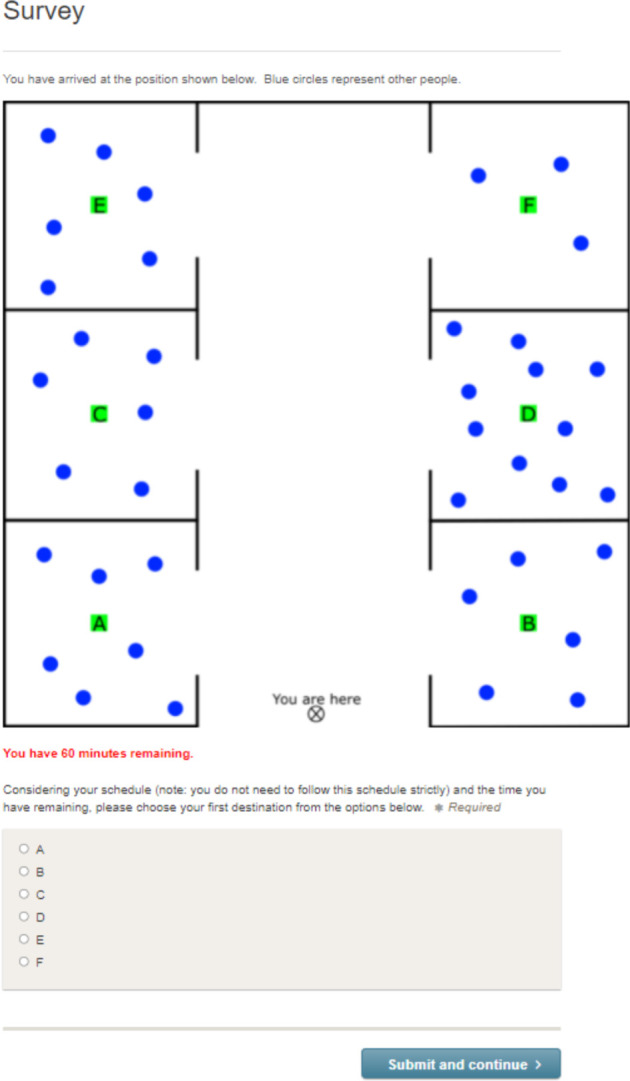
Screenshot of the first question for the schedule conditions. Answering the question would take participants to the next question, which would be in the form shown in figure 13. However, the amount of time remaining, the position of the participant in the image and the answers available would depend on their answer to this question. For non-schedule conditions, there is no mention of the schedule in the question text.

**Figure 13.**
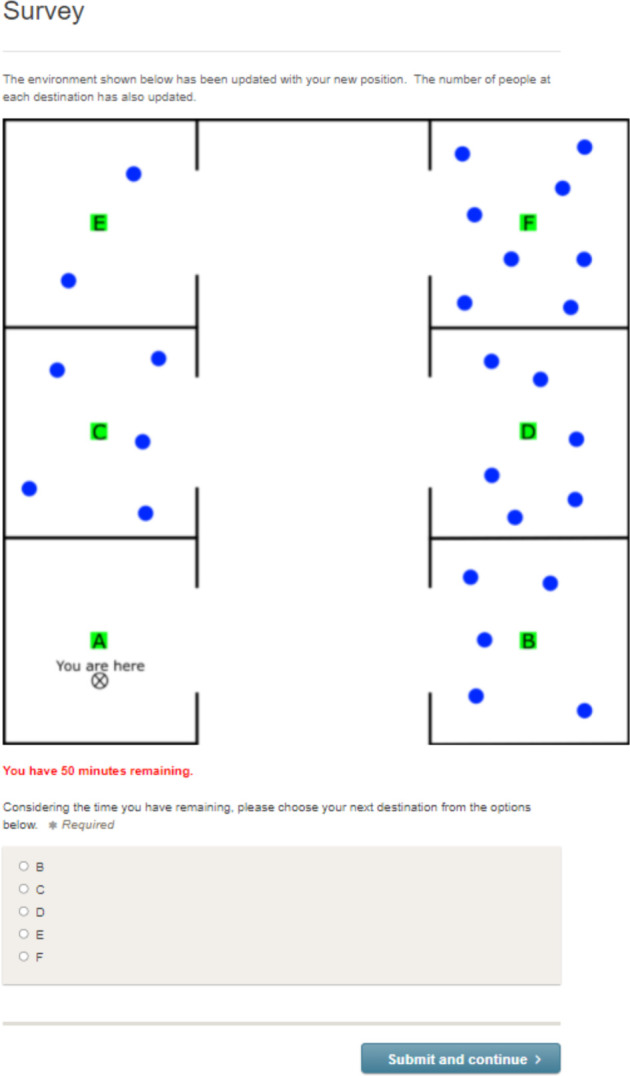
Screenshot of the general form of subsequent questions for the base case and closed conditions. The responses available in subsequent questions depend on the answers to the previous questions. The image would also update for each question answered. The schedule chosen and schedule given conditions would have the same question text as in figure 12. If the participant runs out of hypothetical time, then they are taken to figure 14. Otherwise, the participant proceeds to figure 15 after they answer five questions.

**Figure 14.**
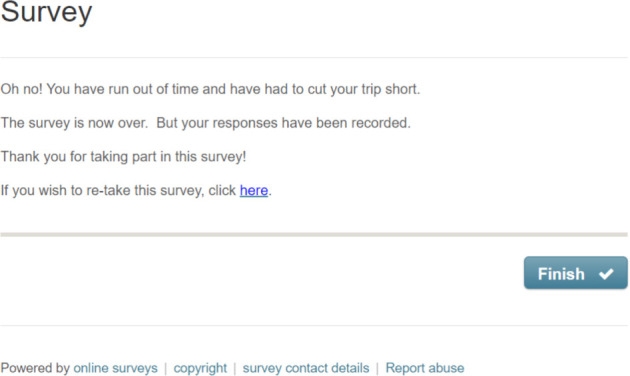
Screenshot of the outcome where participants run out of hypothetical time at any point when answering the preceding questions.

**Figure 15.**
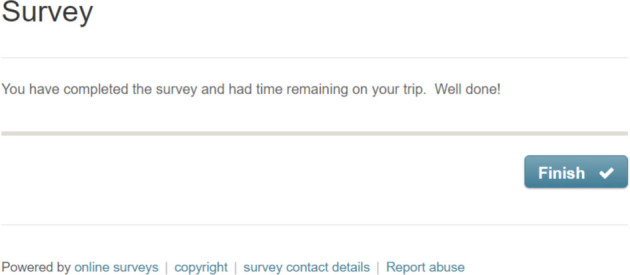
Screenshot of the outcome where participants successfully complete the task.

**Figure 16.**
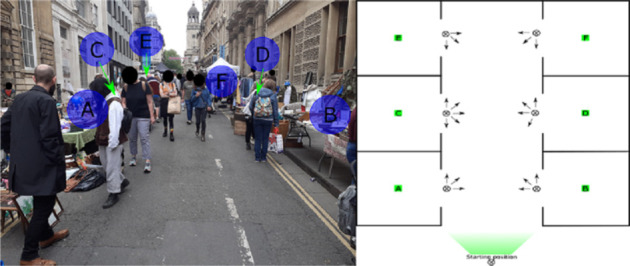
The image was displayed in the broad instructions for the photo condition.

**Figure 17.**
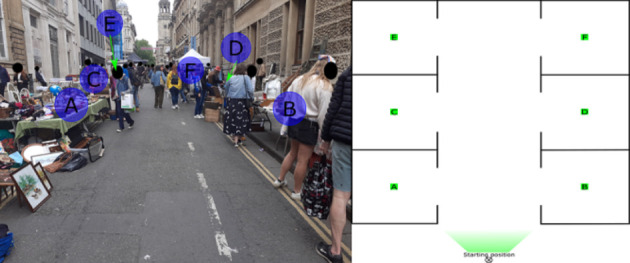
The image was displayed in the specific instructions for the photo condition.

**Figure 18.**
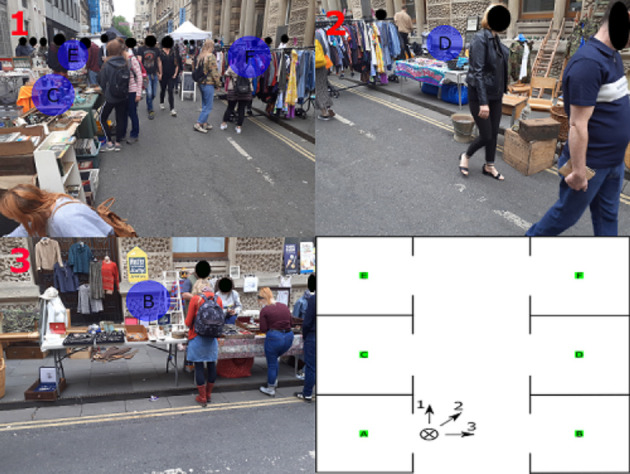
Image in the photo survey when the participant is at destination A.

**Figure 19.**
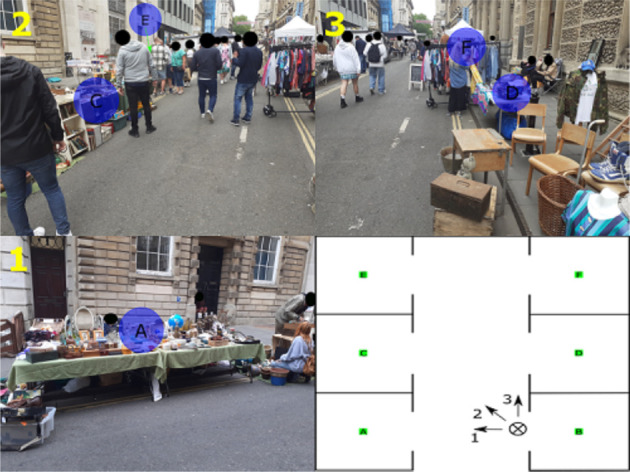
Image in the photo survey when the participant is at destination B.

**Figure 20.**
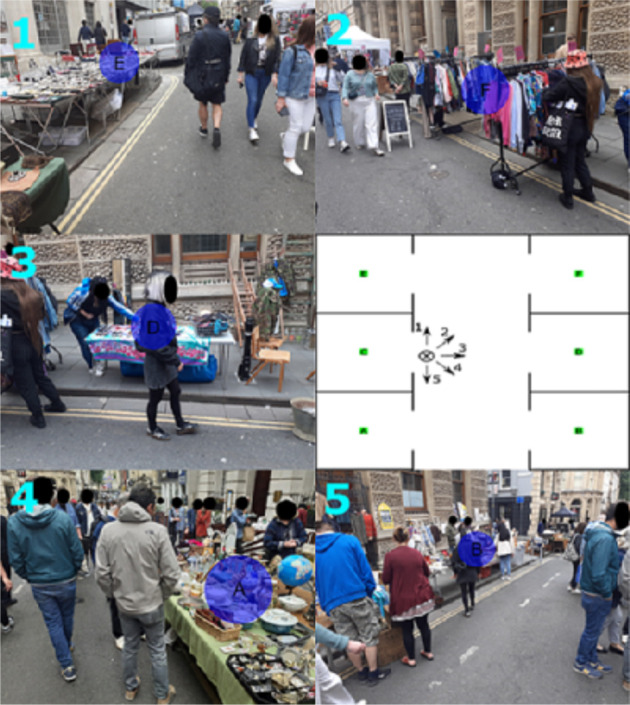
Image in the photo survey when the participant is at destination C.

**Figure 21.**
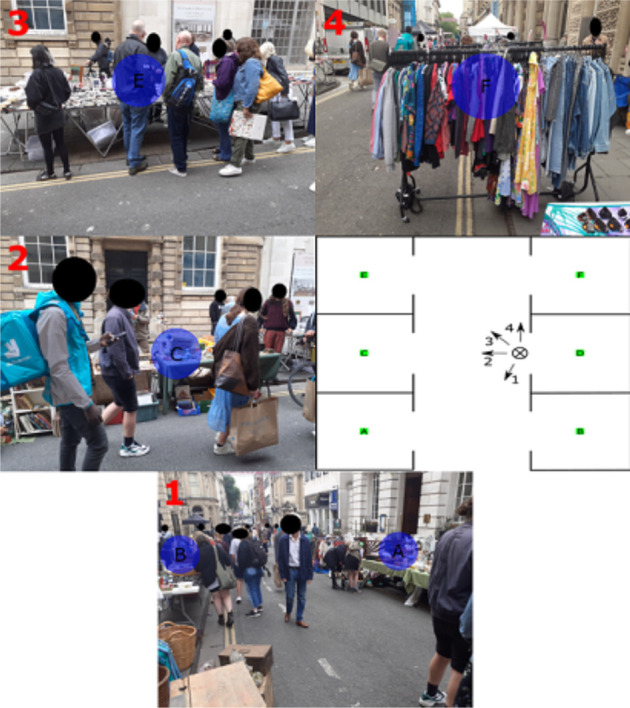
Image in the photo survey when the participant is at destination D.

**Figure 22.**
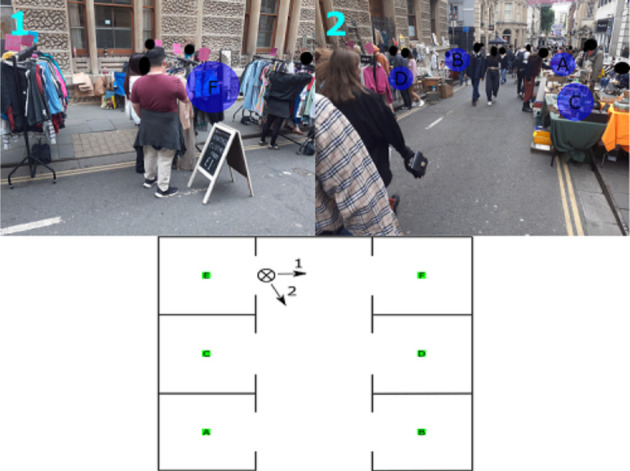
Image in the photo survey when the participant is at destination E.

**Figure 23.**
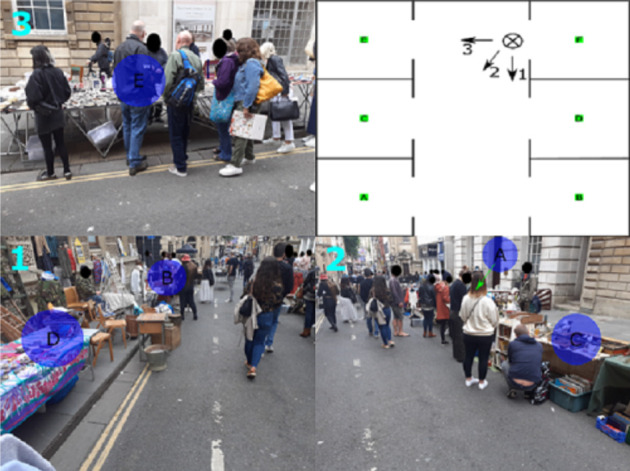
Image in the photo survey when the participant is at destination F.

## Data Availability

The data that has been used in this paper are publicly available: University of Bristol Remote Data Storage Facility (https://doi.org/10.5523/bris.249d43dprgg8x2td33cdmg8kax) [37]. The datasets supporting this article have been uploaded as part of the electronic supplementary material [38].

## Footnotes

1 www.onlinesurveys.ac.uk, accessed on 19 July 2021.

2 www.linkedin.com and www.researchgate.net, both accessed on 19 July 2021.

## Appendix A. Distance and occupancy values

See tables 9–12.

**Table 9.** Distance table for the open and photo environments. The first row represents the distance of each destination from the participant's initial position.

current position	A	B	C	D	E	F
start	1	1	5	5	10	10
A	0	5	3	6	8	10
B	5	0	6	4	10	8
C	3	6	0	5	4	6
D	6	4	5	0	6	4
E	8	10	4	6	0	5
F	10	8	6	4	5	0

**Table 10.** Distance table for the closed environment. The first row represents the distance of each destination from the participant's initial position.

current position	A	B	C	D	E	F
start	2	6	3	5	10	10
A	0	6	6	4	9	9
B	6	0	4	4	8	8
C	6	4	0	4	8	8
D	4	4	4	0	5	5
E	9	8	8	5	0	4
F	9	8	8	5	4	0

**Table 11.** Occupancy table for the open and closed environments.

choice number	A	B	C	D	E	F
1	7	6	6	11	6	3
2	10	5	3	6	2	8
3	3	12	8	3	7	5
4	7	2	11	5	3	13
5	8	9	7	6	9	7

**Table 12.** Occupancy table for the photo environment.

current position	A	B	C	D	E	F
start	1	4	4	1	0	1
A	0	3	2	0	2	3
B	0	0	1	0	4	2
C	2	2	0	2	0	2
D	2	4	1	0	6	0
E	2	1	0	4	0	4
F	3	3	2	0	6	0

## Appendix B. Distance and occupancy weights

See tables 13–15.

**Table 13.** Distance and occupancy weights, *w_d_*(*j*) and *w_d_*(*j*), respectively, for the open environment.

choice number, *j*	*w_d_*(*j*)	*w_o_*(*j*)
1	2	1
2	2	1
3	2	1
4	2	2
5	0.5	0.5

**Table 14.** Distance and occupancy weights, *w_d_*(*j*) and *w_d_*(*j*), respectively, for the closed environment.

choice number, *j*	*w_d_*(*j*)	*w_o_*(*j*)
1	2	1
2	2	1
3	2	1
4	2	1
5	0.5	0.5

**Table 15.** Distance and occupancy weights, *w_d_*(*j*) and *w_d_*(*j*), respectively, for the photo environment.

choice number, j	$w_{d}(j)$	$w_{o}(j)$
1	0.5	0.5
2	0.5	0.5
3	0.25	0.25
4	0.25	0.5
5	0.25	0.5

## Appendix C. Survey screenshots

This appendix contains screenshots of how participants perceived the schedule chosen survey. This acts as a representative example for the information presented in the other surveys. Any differences in the other experimental conditions are discussed in the captions, where appropriate.

See figures 6–15.

## Appendix D. Images in photo condition

See figures 16–23.
